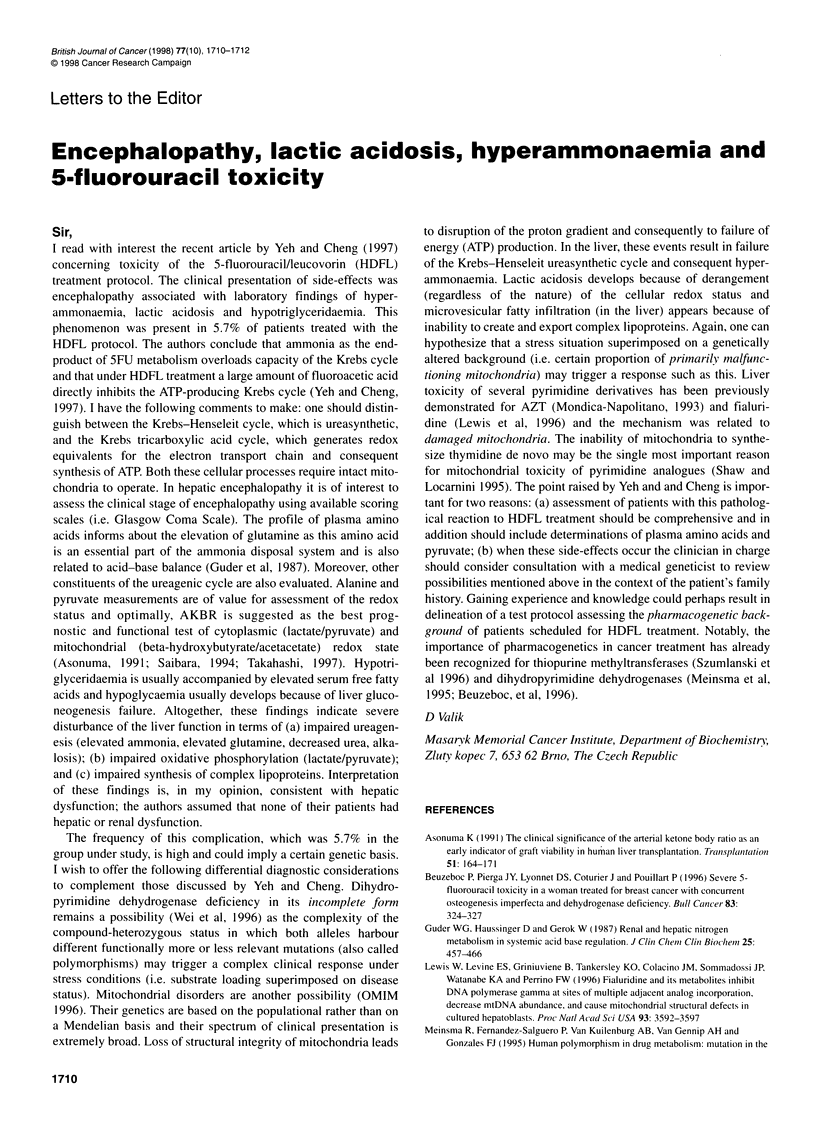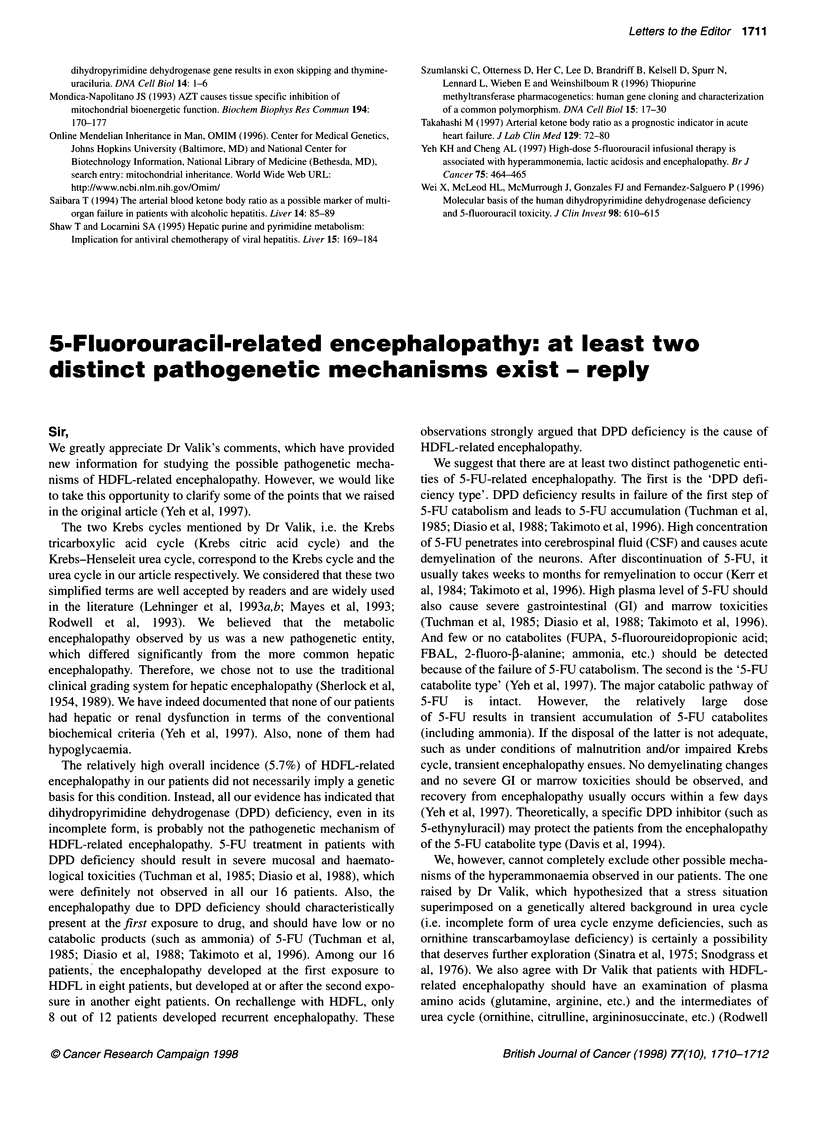# Encephalopathy, lactic acidosis, hyperammonaemia and 5-fluorouracil toxicity.

**DOI:** 10.1038/bjc.1998.282

**Published:** 1998-05

**Authors:** D. Valik


British Journal of Cancer (1998) 77(10), 1710–1712
© 1998 Cancer Research Campaign

Letters to the Editor

### Encephalopathy, lactic acidosis, hyperammonaemia and 5-fluorouracil toxicity

**Sir,**

I read with interest the recent article by Yeh and Cheng (1997) concerning toxicity of the 5-fluorouracil/leucovorin (HDFL) treatment protocol. The clinical presentation of side-effects was encephalopathy associated with laboratory findings of hyperammonaemia, lactic acidosis and hypotriglyceridaemia. This phenomenon was present in 5.7% of patients treated with the HDFL protocol. The authors conclude that ammonia as the end-product of 5FU metabolism overloads capacity of the Krebs cycle and that under HDFL treatment a large amount of fluoroacetic acid directly inhibits the ATP-producing Krebs cycle (Yeh and Cheng, 1997). I have the following comments to make: one should distinguish between the Krebs–Henseleit cycle, which is ureasynthetic, and the Krebs tricarboxylic acid cycle, which generates redox equivalents for the electron transport chain and consequent synthesis of ATP. Both these cellular processes require intact mitochondria to operate. In hepatic encephalopathy it is of interest to assess the clinical stage of encephalopathy using available scoring scales (i.e. Glasgow Coma Scale). The profile of plasma amino acids informs about the elevation of glutamine as this amino acid is an essential part of the ammonia disposal system and is also related to acid–base balance (Guder et al, 1987). Moreover, other constituents of the ureagenic cycle are also evaluated. Alanine and pyruvate measurements are of value for assessment of the redox status and optimally, AKBR is suggested as the best prognostic and functional test of cytoplasmic (lactate/pyruvate) and mitochondrial (beta-hydroxybutyrate/acetacetate) redox state (Asonuma, 1991; Saibara, 1994; Takahashi, 1997). Hypotriglyceridaemia is usually accompanied by elevated serum free fatty acids and hypoglycaemia usually develops because of liver gluconeogenesis failure. Altogether, these findings indicate severe disturbance of the liver function in terms of (a) impaired ureagenesis (elevated ammonia, elevated glutamine, decreased urea, alkalosis); (b) impaired oxidative phosphorylation (lactate/pyruvate); and (c) impaired synthesis of complex lipoproteins. Interpretation of these findings is, in my opinion, consistent with hepatic dysfunction; the authors assumed that none of their patients had hepatic or renal dysfunction.

The frequency of this complication, which was 5.7% in the group under study, is high and could imply a certain genetic basis. I wish to offer the following differential diagnostic considerations to complement those discussed by Yeh and Cheng. Dihydropyrimidine dehydrogenase deficiency in its *incomplete form* remains a possibility (Wei et al, 1996) as the complexity of the compound-heterozygous status in which both alleles harbour different functionally more or less relevant mutations (also called polymorphisms) may trigger a complex clinical response under stress conditions (i.e. substrate loading superimposed on disease status). Mitochondrial disorders are another possibility (OMIM 1996). Their genetics are based on the populational rather than on a Mendelian basis and their spectrum of clinical presentation is extremely broad. Loss of structural integrity of mitochondria leads to disruption of the proton gradient and consequently to failure of energy (ATP) production. In the liver, these events result in failure of the Krebs–Henseleit ureasynthetic cycle and consequent hyperammonaemia. Lactic acidosis develops because of derangement (regardless of the nature) of the cellular redox status and microvesicular fatty infiltration (in the liver) appears because of inability to create and export complex lipoproteins. Again, one can hypothesize that a stress situation superimposed on a genetically altered background (i.e. certain proportion of *primarily malfunctioning mitochondria*) may trigger a response such as this. Liver toxicity of several pyrimidine derivatives has been previously demonstrated for AZT (Mondica-Napolitano, 1993) and fialuridine (Lewis et al, 1996) and the mechanism was related to *damaged mitochondria*. The inability of mitochondria to synthesize thymidine de novo may be the single most important reason for mitochondrial toxicity of pyrimidine analogues (Shaw and Locarnini 1995). The point raised by Yeh and and Cheng is important for two reasons: (a) assessment of patients with this pathological reaction to HDFL treatment should be comprehensive and in addition should include determinations of plasma amino acids and pyruvate; (b) when these side-effects occur the clinician in charge should consider consultation with a medical geneticist to review possibilities mentioned above in the context of the patient's family history. Gaining experience and knowledge could perhaps result in delineation of a test protocol assessing the *pharmacogenetic background* of patients scheduled for HDFL treatment. Notably, the importance of pharmacogenetics in cancer treatment has already been recognized for thiopurine methyltransferases (Szumlanski et al 1996) and dihydropyrimidine dehydrogenases (Meinsma et al, 1995; Beuzeboc, et al, 1996).

*D Valik*

*Masaryk Memorial Cancer Institute, Department of Biochemistry, Zluty kopec 7, 653 62 Brno, The Czech Republic*

**REFERENCES**

Asonuma K (1991) The clinical significance of the arterial ketone body ratio as an early indicator of graft viability in human liver transplantation. *Transplantation* **51**: 164–171

Beuzeboc P, Pierga JY, Lyonnet DS, Coturier J and Pouillart P (1996) Severe 5-fluorouracil toxicity in a woman treated for breast cancer with concurrent osteogenesis imperfecta and dehydrogenase deficiency. *Bull Cancer* **83**: 324–327

Guder WG, Haussinger D and Gerok W (1987) Renal and hepatic nitrogen metabolism in systemic acid base regulation. *J Clin Chem Clin Biochem* **25**: 457–466

Lewis W, Levine ES, Griniuviene B, Tankersley KO, Colacino JM, Sommadossi JP, Watanabe KA and Perrino FW (1996) Fialuridine and its metabolites inhibit DNA polymerase gamma at sites of multiple adjacent analog incorporation, decrease mtDNA abundance, and cause mitochondrial structural defects in cultured hepatoblasts. *Proc Natl Acad Sci USA* **93**: 3592–3597

Meinsma R, Fernandez-Salguero P, Van Kuilenburg AB, Van Gennip AH and Gonzales FJ (1995) Human polymorphism in drug metabolism: mutation in the dihydropyrimidine dehydrogenase gene results in exon skipping and thymine-uraciluria. *DNA Cell Biol* **14**: 1–6
Mondica-Napolitano JS (1993) AZT causes tissue specific inhibition of mitochondrial bioenergetic function. *Biochem Biophys Res Commun* **194**: 170–177
Online Mendelian Inheritance in Man, OMIM (1996). Center for Medical Genetics, Johns Hopkins University (Baltimore, MD) and National Center for Biotechnology Information, National Library of Medicine (Bethesda, MD), search entry: mitochondrial inheritance. World Wide Web URL: http://www.ncbi.nlm.nih.gov/Omim/
Saibara T (1994) The arterial blood ketone body ratio as a possible marker of multi-organ failure in patients with alcoholic hepatitis. *Liver* **14**: 85–89
Shaw T and Locarnini SA (1995) Hepatic purine and pyrimidine metabolism: Implication for antiviral chemotherapy of viral hepatitis. *Liver* **15**: 169–184
Szumlanski C, Otterness D, Her C, Lee D, Brandriff B, Kelsell D, Spurr N, Lennard L, Wieben E and Weinshilboum R (1996) Thiopurine methyltransferase pharmacogenetics: human gene cloning and characterization of a common polymorphism. *DNA Cell Biol* **15**: 17–30
Takahashi M (1997) Arterial ketone body ratio as a prognostic indicator in acute heart failure. *J Lab Clin Med* **129**: 72–80
Yeh KH and Cheng AL (1997) High-dose 5-fluorouracil infusional therapy is associated with hyperammonemia, lactic acidosis and encephalopathy. *Br J Cancer* **75**: 464–465
Wei X, McLeod HL, McMurrough J, Gonzales FJ and Fernandez-Salguero P (1996) Molecular basis of the human dihydropyrimidine dehydrogenase deficiency and 5-fluorouracil toxicity. *J Clin Invest* **98**: 610–615